# Binding of Cationic Bis-porphyrins Linked with *p*- or *m*- Xylylenediamine and Their Zinc(II) Complexes to Duplex DNA

**DOI:** 10.3390/molecules13123117

**Published:** 2008-12-15

**Authors:** Yoshinobu Ishikawa, Naoki Yamakawa, Tadayuki Uno

**Affiliations:** 1 School of Pharmaceutical Sciences, University of Shizuoka, 52-1 Yada, Suruga-ku, Shizuoka 422-8526, Japan; 2 Graduate School of Pharmaceutical Sciences, Kumamoto University, 5-1 Oe-honmachi, Kumamoto 862-0973, Japan; E-mail: bananao@gpo.kumamoto-u.ac.jp (N. Y.); 3 Graduate School of Pharmaceutical Sciences, Osaka University, 1-6 Yamadaoka, Suita, Osaka 565-0871, Japan

**Keywords:** Porphyrin, DNA, Binding mode, Zinc, Titration, Circular dichroism, Viscometry, Distamycin, Molecular docking simulation

## Abstract

Spectroscopic, viscometric, and molecular docking analysis of binding of cationic bis-porphyrins linked with *p*- or *m*-xylylenediamine (**H_2_pXy** and **H_2_mXy**) and their zinc(II) complexes (**ZnpXy** and **ZnmXy**) to duplex DNA are described. **H_2_pXy** and **H_2_mXy** bound to calf thymus DNA (CTDNA) stronger than unichromophoric **H_2_TMPyP**, and showed exciton-type induced circular dichroism spectra of their Soret bands. The **H_2_TMPyP**-like units of the metal-free bis-porphyrins did not intercalate into CTDNA, and thus the binding mode is outside binding with intramolecular stacking. **ZnpXy** showed favorable binding to A·T over G·C region, and should lie in the major groove of A·T region.

## Introduction

Cationic, water-soluble *meso*-tetrakis(*N*-methyl-4-pyridyl)porphine (**H_2_TMPyP**) and its derivatives have attracted considerable attention in association with their interaction with nucleic acids and the potential medical applications of this [1,2]. The cationic macrocycles tightly bind to nucleic acid duplexes, triplexes and quadruplexes with the following binding modes: intercalation, groove binding, outside binding with self-stacking and external stacking [3,4,5,6,7]. These binding modes have been proposed from spectroscopic, physicochemical, and biochemical analyses [8,9,10,11,12]. The close association of such compounds with a specific gene should cause down-regulation of the gene expression by interfering the binding of regulatory proteins to the gene. The dyes can also cleave DNA on irradiation with visible light [13,14,15,16]. Irreversible DNA damages in malignant cells are expected to disturb genetic events, and thus to suppress the proliferation of the malignant cells. Hence, **H_2_TMPyP** and their derivatives showing the bifunctionality of the DNA binding and cleaving ability have considerable potential to be useful anti-tumor drugs [17,18].

**Figure 1:**
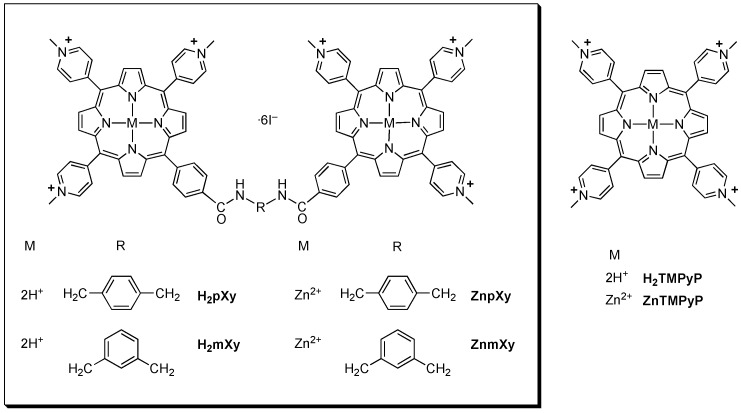
Cationic porphyrins in this study.

We have recently reported the synthesis of cationic bis-porphyrins (**H_2_pXy** and **H_2_mXy**) and their zinc(II) complexes (**ZnpXy** and **ZnmXy**) with two **H_2_TMPyP**-like chromophores bridged by *p*- or *m*-xylylenediamine with the purpose of developing effective DNA photocleaving agents [19]. The xylylene linkers and zinc ion were introduced to control interchromophoric interaction that is involved in photosensitization of the cationic bis-porphyrins. The molar absorptivities of all the bis-porphyrins in aqueous solution remained unchanged over a wide range of concentrations, indicating the absence of self-aggregation properties. In particular, the molar absorptivity of the zinc(II) complex of the *p*‑xylylenediamine-linked bis-porphyrin in aqueous solution was 2.0 times as large as that of unichromophoric **ZnTMPyP**, suggesting the absence of both intermolecular and intramolecular interchromophoric interactions. The metal-free *p*-xylylenediamine-linked bis-porphyrin showed a more efficient conversion ability of supercoiled to nicked circular pUC18 plasmid DNA by photosensitization than the metal-free *m*-xylylenediamine-linked one. Furthermore, the zinc complexes of the bis-porphyrins exhibited more potent DNA photocleavage than did the metal-free bis-porphyrins. Singlet oxygen productivity of the four cationic bis-porphyrins was determined by measuring the decomposition rate of 1,3-diphenylisobenzofuran. The amount of singlet oxygen generated by photosensitization of the zinc(II) complex of the *p*-xylylenediamine-linked bis-porphyrin in aqueous solution was 2.1 times as large as **ZnTMPyP**, indicating the singlet oxygen productivity. A strict correlation between the DNA photocleaving abilities and singlet oxygen productivities of the cationic porphyrins in aqueous solution was found. 

In this paper, we describe the spectroscopic analysis of binding of the cationic bis-porphyrins linked with *p*- or *m*-xylylenediamine and their zinc(II) complexes to duplex DNA, the viscometric analysis of **H_2_pXy**- and **H_2_mXy**-CTDNA complexes, and a molecular docking analysis of **ZnpXy**-[d(CGCAAATTTGCG)]_2_ complex. These bis-porphyrins bound to calf thymus DNA (CTDNA) stronger than unichromophoric **H_2_TMPyP** and **ZnTMPyP**. Versatile binding modes for the bis-porphyrins were revealed, depending on the difference in the linker part and the presence of zinc(II) ion at the core. In particular, **ZnpXy** showed favorable binding to A·T over G·C region, and should lie in the major groove of A·T region. Our finding should contribute to the development of anti-tumor drugs applied not only in photodynamic therapy but also in chemotherapy.

## Results

### Visible spectra

**Figure 2:**
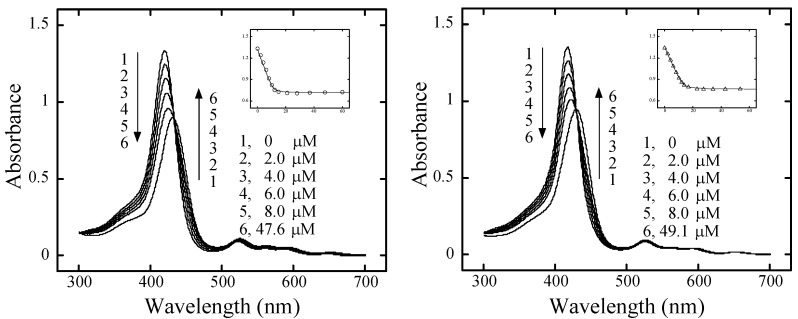
(**a**, left) Absorption spectral change of **H_2_pXy** (4.9 μM) with addition of CTDNA and absorbance change at 419 nm, (**b**, right) absorption spectral change of **H_2_mXy** (4.9 μM) with addition of CTDNA and absorbance change at 419 nm. Theoretical solid lines were also drawn with the *K* and *n* values in Table 1.

Thebinding process of the cationic bis-porphyrins to CTDNA was monitored by visible absorption spectroscopy. A buffered solution of the cationic bis-porphyrin was titrated with a stock solution of CTDNA. For the metal-free **H_2_pXy** and **H_2_mXy** (4.9 μM each), intensity of *λ*_max_^Soret^ decreased monotonously by adding CTDNA with one set of isosbestic points, as shown in Figure 2a for **H_2_pXy** and Figure 2b for **H_2_mXy**, respectively. Figure 2a and Figure 2b also show the correponsing absorbance changes of **H_2_pXy** at 419 nm and **H_2_mXy** at 417 nm with addition of CTDNA.

Along with reduction of the intensities, *λ*_max_^Soret^ of both **H_2_pXy** and **H_2_mXy** shifted to longer wavelengths. Bathochromic shifts (Δ*λ*) and hypochromicities (*H*) for **H_2_pXy** and **H_2_mXy** are listed in Table 1. Because these metal-free cationic bis-porphyrins were monomeric below 70 μM of CTDNA, and isosbestic points were shown in their spectral changes, the binding constant (*K*) and the number of binding sites per base pair (*n*) were estimated by the Scatchard analysis [3]. The values of *K* and *n* for **H_2_pXy** and **H_2_mXy** are also listed in Table 1. Theoretical curves with these values reproduced well the absorbance changes at their Soret maxima, as shown in Figure 2a for **H_2_pXy** and Figure 2b for **H_2_mXy**, respectively.

**Figure 3:**
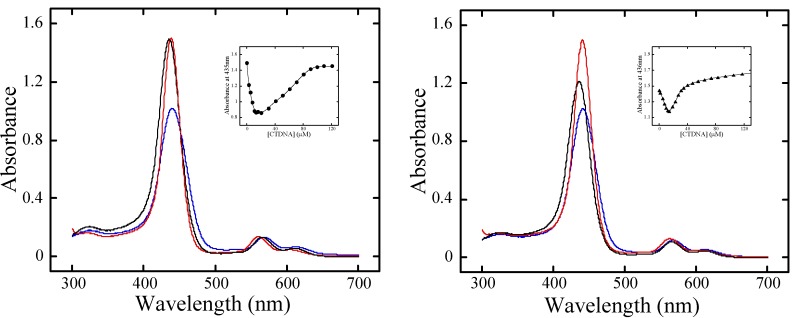
(**a**, left) Absorption spectral change of **ZnpXy** (3.5 μM) with addition of CTDNA (black, 0 μM; blue, 12.9 μM; red, 128 μM) and absorbance change at 435 nm, (**b**, right) absorption spectral change of **ZnpXy** (3.5 μM) with addition of CTDNA (black, 0 μM; blue, 7.7 μM; red, 121 μM) and absorbance change at 436 nm.

**Table 1:** Spectroscopic data for cationic porphyrins bound to CTDNA in the buffer^A^.

	Δ*λ* (nm)^B^	*H* (%)^C^	*K* (μM^–1^)	*n*	Δ*ε* (M^–1^cm^–1^)	(nm)^D^
**H_2_pXy**	12	36	14.2	0.43	+75 (410)	–50 (428)
**H_2_mXy**	10	31	9.8	0.38	+28 (411)	–25 (430)
**H_2_TMPyP^E^**	10	46	4.3	0.33	+6 (418)	–13 (433)
**ZnpXy**	2	–4	-	-	+54 (415)	+28 (430)
**ZnmXy**	3	–23	-	-	+31 (414)	+19 (434)
**ZnTMPyP^F^**	2	–4	-	-	+6 (416)	+12 (433)

^A^ Buffer: 10 mM sodium phosphate and 0.1M NaCl (pH 7.0); ^B^: bathochromic shift; ^C^ *H*: Hypochromicity was determined by following equation: *H* = (A_f_ – A_b_)/A_f_ ×100, where A_f_ and A_b_ represent the absorbance of the Soret maximum of free and bound porphyrins, respectively (a negative value indicates hyperchromicity); ^D^: Molar circular-dichroic absorption of induced CD peaks at Soret band at *R* = 0.01; ^E^: reference [14]; ^F^: reference [15].

In contrast to the monotonous spectral change of the metal-free bis-porphyrins, the spectral behavior of the zinc complexes upon addition of CTDNA was complex. The spectral change and absorbance change at 435 nm for **ZnpXy** (3.3 μM) titrated with CTDNA is shown Figure 3a. Intensity of the *λ*_max_^Soret^ decreased, with a slight red shift at the early stage (*R* ( = [porphyrin] / [base pairs]) ≥ 0.43), and then increased with further DNA addition. The final intensity of slight red-shifted *λ*_max_^Soret^ was almost the same with the initial intensity (­Table 1). Figure 3b shows the spectral change and absorbance change at 433 nm for **ZnmXy** (4.7 μM) with addition of CTDNA. The spectral behavior was also not simple. Notably, a moderate hyperchromicity was observed for **ZnmXy** (–23%, Table 1). In either case we were unable to estimatd *K* and *n* values for these zinc complexes because of their irregular absorbance changes.

### Induced circular dichroism (iCD)

To clarify the binding modes of the cationic bis-porphyrins, iCD of the bis-porphyrins in the presence of DNA were recorded. Whereas all four cationic bis-porphyrins showed no iCD spectrum in their Soret regions in the absence of DNA, characteristic CD spectra were induced in the presence of duplex DNA.

**Figure 4:**
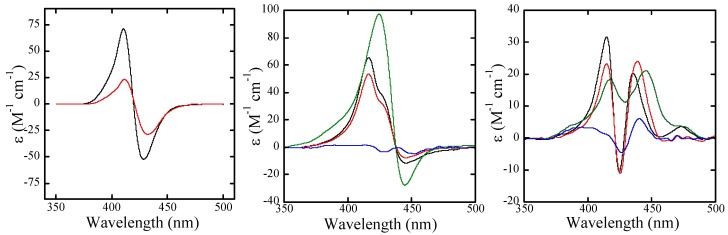
(**a**, left) iCD spectra of **H_2_pXy** (black) and **H_2_mXy** (red) at their Soret bands in the presence of CTDNA at *R* = 0.01, (**b**, center) iCD spectra of **ZnpXy** in the presence of CTDNA without distamycin (black) and with distamycin (red, [distamycin] / [base pairs] = 0.6), poly(dA)·poly(dT) (green), and poly(dG)·poly(dC) (blue) at *R* = 0.01, (**c**, right) iCD spectra of **ZnpXy** in the presence of CTDNA without distamycin (black) and with distamycin (red, [distamycin] / [base pairs] = 0.6), poly(dA)·poly(dT) (green), and poly(dG)·poly(dC) (blue) at *R* = 0.01.

For metal-free **H_2_pXy** and **H_2_mXy**, excitonic spectra were observed at *R* = 0.01 in the buffer, as shown in Figure 4a. The molar ellipticity of the **H_2_pXy**-CTDNA complex was larger than that of **H_2_mXy**-CTDNA (Table 1). On the other hand, a large positive peak at 415 nm, and two positive peaks at 415 nm and minor at 430 nm were revealed for the **ZnpXy**- and the **ZnmXy**-CTDNA complex at *R* = 0.01, respectively (Figure 4b and Figure 4c). A large trough was seen in the case of **ZnmXy**. The band shape of the iCD spectrum for the **ZnpXy**-CTDNA complex did not change, even after addition of distamycin, a well-known minor groove binder. On the other hand, for the **ZnmXy**-CTDNA complex the band shape of the iCD spectrum was changed by the addition of distamycin. The larger peak of the iCD spectrum for **ZnmXy** decreased, and the smaller peak increased by adding distamycin to the **ZnmXy**-CTDNA complex. 

Homogeneous, synthetic polynucleotides, poly(dA)·poly(dT) and poly(dG)·poly(dC), were used to examine sequence specificity of the binding for **ZnpXy** and **ZnmXy**. For **ZnpXy** a large positive peak at 424 nm were observed in the presence of poly(dA)·poly(dT) together with relatively rather small negative peak at 444 nm at *R* = 0.01. Conversely, no iCD was observed in the presence of poly(dG)·poly(dC) at *R* = 0.01, as shown in Figure 4b. In the case of **ZnmXy** two positive peaks at 417 and 445 nm were observed in the presence of poly(dA)·poly(dT) at *R* = 0.01. In the presence of poly(dG)·poly(dC), a weak excitonic spectrum was seen at *R* = 0.01 (Figure 4c). Thus, the binding modes of the metal-free bis-porphyrins, **ZnpXy**, and **ZnmXy** to duplex DNA were clearly different from each other.

### Viscometry

**Figure 5:**
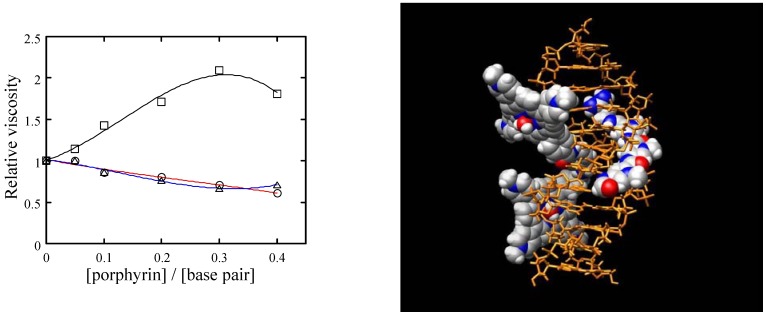
(**a**, left) Plots of the relative viscosities of CTDNA solutions in the presence of **H_2_TMPyP** (open squares), **H_2_pXy** (open circles) and **H_2_mXy** (open triangles) versus *R*, (**b**, right) A predicted docking pose for **ZnpXy** in the major groove of [d(CGCAAATTTGCG)]_2_, along with distamycin in the minor groove.

Viscosity measurements were performed with 200 μM CTDNA in the buffer to obtain more accurate information about the binding mode of metal-free **H_2_pXy** and **H_2_mXy** (Figure 5a). A relative viscosity was increased (1.0 to 2.1) with increase in **H_2_TMPyP** concentration at *R* ≤ 0.3, whereas the relative viscosity decreased with increase in **H_2_pXy** and **H_2_mXy** concentration (1.0 to 0.71 and 0.67, respectively) in the *R* range. These results certainly reflect the substantial difference between their binding modes of **H_2_TMPyP** and the metal-free bis-porphyrins.

### Molecular Modeling

Molecular docking simulation was carried out for **ZnpXy** to predict its binding mode to duplex DNA. The crystal structure of [d(CGCAAATTTGCG)]_2_ with distamycin was selected as a target structure (PDB ID: 2DND) [20], because **ZnpXy** favorably bound to A·T rich sequence, and the binding was not influenced even in the presence of minor groove binder distamycin that binds to A·T-rich sequence. After the distamycin molecule was removed from the crystal structure and then alpha sites were generated on the surface of the major groove of the duplex using the *SITE FINDER* module of MOE [21], the model structure of **ZnpXy** was docked on the surface of the major groove using the *DOCK* module of MOE. A predicted, top-ranking pose of **ZnpXy** along with the duplex and distamycin is shown in Figure 5b. The distamycin molecule interacts with five A·T base pairs of six A·T base pairs in the crystal structure. On the other hand, the predicted docking pose for **ZnpXy** shows one of two porphyrin units and the linker part of **ZnpXy** cover one G·C base pairs and four A·T base pairs of six A·T base pairs. The other porphyrin unit lies on the phosphate diester backbone of the duplex. The molecular length of the docking pose of **ZnpXy** along the groove was roughly two times as long as that of distamycin. 

## Discussion

The aim of this work was to analyze the interaction of cationic bis-porphyrins bridged by *p*- or *m*-xylylenediamine and their zinc(II) complexes with duplex DNA toward the development of DNA-targeting drugs capable controlling gene expression.

With increase in the concentration of CTDNA, the intensity of the Soret bands of metal-free **H_2_pXy** and **H_2_mXy** decreased monotonously with one set of isosbestic points, as shown in Figure 2a and Figure 2b for **H_2_pXy** and **H_2_mXy**, respectively. Their binding steps were found to be single, and the *n* and *K* values were estimated by Scatchard analysis. The *n* values for both **H_2_pXy** and **H_2_mXy** indicate that the four cationic molecules per ten base pairs bind to CTDNA. The binding constants of **H_2_pXy** and **H_2_mXy** were roughly three times and twice as great than that of **H_2_TMPyP**, respectively. Their higher *K* values are probably due to the greater number of positive charges per one molecule of the hexa-cationic bis-porphyrins in comparison with tetra-cationic **H_2_TMPyP**. Notably, their binding constants were independent of their DNA photocleaving activities of pUC18 plasmid DNA [19]. Because more than 99.6% of the cationic bis-porphyrins and **H_2_TMPyP** bind to CTDNA under the condition ([base pair] = 60.0 μM, [porphyrin] = 0.6 μM), their affinities to DNA should not be related to their DNA photocleaving activities. As described in previous paper, the singlet oxygen production and DNA photocleaving activity of the cationic bis-porphyrins by photosensitization in aqueous solution is most likely to be affected by the intramolecular interchromophoric interaction. 

The spectral changes of **H_2_pXy** and **H_2_mXy** with addition of CTDNA were accompanied by the substantial hypochromicities and bathochromic shifts (Table 1). Their binding behaviors were similar to that of intercalating **H_2_TMPyP** in terms of showing monotonous decrease in their *λ*_max_^Soret^ with isosbestic points [14]. However, it is not distinguishable from these results whether the **H_2_TMPyP**-like chromophores of the metal-free bis-porphyrins intercalate between base pairs or not, because intercalated complexes are characterized by substantial hypochromicity of the Soret band (≥ 35%) and large bathochromic shift (≥ 15 nm) [1].

In contrast to the metal-free bis-porphyrins, the binding process of their zinc(II) complexes to CTDNA were not simple (Figure 3a and Figure 3b). The fact that both **ZnpXy** and **ZnmXy** showed the slight red-shifts and not hypochromicity but hyperchromicities at the final stage of their DNA binding indicate that their binding were not by intercalation. The degrees of the red-shift and hyperchromicity for **ZnpXy** were almost identical to those for **ZnTMPyP**, suggesting that the binding of the **ZnTMPyP**-like chromophores of **ZnpXy** to CTDNA should be the same with that of **ZnTMPyP**, and supporting that there is no interchromophoric interaction for **ZnpXy** [19]. On the other hand, the moderate hyperchromicity observed for **ZnmXy** is likely due to the relaxation of the intramolecular interchromophoric interaction in binding to a large amount of CTDNA. 

Induced CD in the Soret region is very helpful for analysis of the binding mode of achiral porphyrins to chiral DNA. For **H_2_pXy** and **H_2_mXy**, bisignate CD spectra were induced in binding to CTDNA (Figure 4a). As reported previously, these metal-free cationic bis-porphyrins showed an intramolecular interchromophoric interaction, and were monomeric in aqueous solution [19]. In addition to the fact, these metal-free bis-porphyrins bound to CTDNA in a single step. Thus, the excitonic spectra suggest the binding mode of the metal-free bis-porphyrins is outside binding with *intramolecular stacking* on the DNA surface. However, intercalative binding could also be another candidate, because the binding processes and iCD spectra for these cationic bis-porphyrins under the buffered condition were similar to those for intercalating **H_2_TMPyP** [14]. To distinguish these binding modes, we carried out the viscosity measurements. The relative viscosity of CTDNA increased with increase in the concentration of **H_2_TMPyP** at *R* ≤ 0.3 (Figure 5a), indicating that **H_2_TMPyP** does intercalate between the base pairs of CTDNA [22]. On the other hand, the relative viscosity did not increase in the case of **H_2_pXy** and **H_2_mXy**. On the basis of these results, we concluded that the binding mode of **H_2_pXy** and **H_2_mXy** to CTDNA is outside binding with intramolecular stacking. 

The iCD spectrum of the **ZnpXy**-CTDNA complex at *R* = 0.01 showed a large positive peak in the Soret region (Figure 4b), which was similar to that of the **ZnTMPyP**-CTDNA complex [15]. The five-coordinate **ZnTMPyP**-like chromophores of **ZnpXy** should not interact with each other at *R* = 0.01, as deduced from the visible titration data. The band shape of the iCD spectrum of **ZnpXy**-CTDNA complex was independent of the presence of a minor groove binder distamycin that favors A·T-rich regions in DNA. In addition, **ZnpXy** was found to prefer A·T to G·C sequence, revealed by the iCD data in the presence of the synthetic polynucleotides, poly(dA)·poly(dT) and poly(dG)·poly(dC). Accordingly, the binding mode of **ZnpXy** to CTDNA is most likely to be major groove binding. The zinc(II) insertion into **H_2_pXy** resulted in the clear-cut binding mode transition. On the other hand, the iCD spectrum of the **ZnmXy**-CTDNA complex at *R* = 0.01 showed two positive peaks and a large trough with negative sign between the peaks (Figure 4c). The visible titration result for **ZnmXy** in binding to CTDNA suggests the intramolecular interaction for **ZnmXy** was not completely relaxed even in the presence of an excess of CTDNA. Hence, the binding mode of **ZnmXy** to CTDNA should be groove binding with intramolecular interaction. Unlike **ZnpXy**, **ZnmXy** is likely to bind to not only major but also minor groove of CTDNA, because the band shape of the iCD spectrum changed in the presence of distamycin [23]. Sequence selectivity of **ZnmXy** was lower than that of **ZnpXy**.

Because **ZnpXy** was found to prefer A·T region and bind to major groove of CTDNA, we carried out a molecular docking simulation to model the 3D structure of **ZnpXy**-[d(CGCAAATTTGCG)]_2_ complex. The top-ranking pose of **ZnpXy** suggests one of two porphyrin units and the linker part of **ZnpXy** contact on the surface of the major groove of the A·T region (Figure 5b). On the other hand, the other porphyrin unit does not contact on the surface of the G·C region, and lies on the phosphate diester backbone of the G·C region in this model. This result is reasonable because most DNA-interactive drugs do not bind to the interior of the major groove of G·C region of duplex DNA. Because most DNA-binding proteins like transcription factors interact with duplex DNA in the major groove [24], major groove binding molecules such as **ZnpXy** could be effective drugs capable of controlling gene expression. 

## Conclusions

We have demonstrated the spectroscopic, viscometric and molecular modeling analyses of binding of the metal-free cationic bis-porphyrins, **H_2_pXy** and **H_2_mXy**, linked with *p*- or *m*-xylylenediamine, and their zinc(II) complexes, **ZnpXy** and **ZnmXy**, to duplex DNA. These bis-porphyrins are found to bind to CTDNA stronger than unichromophoric **H_2_TMPyP** and **ZnTMPyP**, which is most likely to be due to the increase in the positive charges per molecule. Versatile binding processes were observed for the bis-porphyrins, depending on the difference in the linker part and the presence of zinc(II) ion at the cores. Interestingly, **ZnpXy** showed preferential binding to A·T over G·C region, and is most likely to be in the major groove. Thus, suitable choice of linkers and metals would lead to binding specificity of cationic oligo-porphyrins to duplex DNA. 

## Experimental Section

### Materials

The metal-free bis-porphyrins and the zinc(II) complexes were synthesized as reported previously [19]. The tosylate salt of **H_2_TMPyP** was purchased from Dojin Chemical Co. CTDNA and the synthetic DNA, poly(dA)·poly(dT) and poly(dG)·poly(dC), were purchased from Sigma Chemical Co. The aqueous solution was quantitated spectrophotometrically using *ε*_260_ = 13,200 M(base pairs)^–1^·cm^–1^ for CTDNA, *ε*_260_ = 12,000 M(base pairs)^–1^·cm^–1^ for poly(dA)·poly(dT), and *ε*_253_ = 14,800 M(base pairs)^–1^·cm^–1^ for poly(dG)·poly(dC). Unless otherwise noted, a buffered solution consisted of 10 mM sodium phosphate and 100 mM NaCl (pH 7.0).

### Spectral measurements

Aliquots of CTDNA solution were added to a cationic bis-porphyrin solution (ca. 4.0 μM), and the spectral measurements were performed at 25 ˚C in the buffer. The binding constant (*K*) and the number of binding sites per base pair (*n*) were estimated from the spectral changes for the metal-free cationic bis-porphyrins, following Scatchard analysis [3]. The iCD spectra of a cationic bis-porphyrin were recorded on a JASCO J-720 spectropolarimeter after addition of DNA to a solution of the cationic bis-porphyrin. Twenty independent spectra were averaged and smoothed. 

### Viscosity studies

Viscosity measurements were done using an Ubbelohde viscometer in a thermostatted water bath at 25±0.1 ˚C. The flow times of 1.0 mL of the buffered solution and then 1.0 mL of the buffered solution of 200 μM CTDNA were recorded. Subsequently, the flow times of the solution of 200 μM CTDNA with a porphyrin at various concentration ratios were recorded. At least three trials were done with a deviation of less than ±0.5 s for each measurement.

### Molecular modeling

The structural model of **ZnpXy** was built using the *BUILDER* module of MOE [21]. The structure of the porphyrin core was optimized in MOPAC. The crystal structure of the [d(CGCAAATTTGCG)]_2_ with a distamycin molecule was selected for the receptor molecule. The parameters and charges were assigned with MMFF94x force field. Water and distamycin molecules around the duplex were removed, and hydrogen atoms were added. After alpha-site spheres were generated in the interior of the major groove using the *SITE FINDER* module of MOE, the structural model of **ZnpXy** were docked on the surface of the interior of the major groove using the *DOCK* module of MOE. 

## References

[1] Pasternack R.F., Gibbs E.J. (1996). Porphyrin and metalloporphyrin interactions with nucleic acids. Met Ions Biol. Syst..

[2] Pratviel G., Bernadou J., Meunier B. (1996). Selective DNA cleavage by metalloporphyrin derivatives. Met. Ions Biol. Syst..

[3] Uno T., Hamasaki K., Tanigawa M., Shimabayashi S. (1997). Binding of *meso*-Tetrakis(N-methylpyridinium-4-yl)porphyrin to Double Helical RNA and DNA·RNA Hybrids. Inorg. Chem..

[4] Kim J.O., Lee Y.A., Yun B.H., Han S.W., Kwag S.T., Kim S.K. (2004). Binding of meso-tetrakis(N-methylpyridinium-4-yl)porphyrin to AT oligomers: effect of chain length and the location of the porphyrin stacking. Biophys. J..

[5] Evans S.E., Mendez M.A., Turner K.B., Keating L.R., Grimes R.T., Melchoir S., Szalai V.A. (2007). End-stacking of copper cationic porphyrins on parallel-stranded guanine quadruplexes. J. Biol. Inorg. Chem..

[6] Han H., Langley D.R., Rangan A., Hurley L.H. (2001). Selective interactions of cationic porphyrins with G-quadruplex structures. J. Am. Chem. Soc..

[7] Yamashita T., Uno T., Ishikawa Y. (2005). Stabilization of guanine quadruplex DNA by the binding of porphyrins with cationic side arms. Bioorg. Med. Chem..

[8] Seenisamy J., Rezler E.M., Powell T.J., Tye D., Gokhale V., Joshi C.S., Siddiqui-Jain A., Hurley L.H. (2004). The dynamic character of the G-quadruplex element in the c-MYC promoter and modification by TMPyP4. J. Am. Chem. Soc..

[9] Ohyama T., Mita H., Yamamoto Y. (2005). Binding of 5,10,15,20-tetrakis(N-methylpyridinium-4-yl)- 21H,23H-porphyrin to an AT-rich region of a duplex DNA. Biophys. Chem..

[10] Park T.G., Ko J.H., Ryoo A.Y., Kim J.M., Cho D.W., Kim S.K. (1760). Binding modes of V(=O)meso-tetrakis(N-methylpyridinium-4-yl)porphyrin to various synthetic DNAs studied by polarized spectroscopy. Biochim. Biophys. Acta.

[11] Nov J., Urbanová M. (2007). Vibrational and electronic circular dichroism study of the interactions of cationic porphyrins with (dG-dC)_10_ and (dA-dT)_10_. Biopolymers.

[12] Uno T., Aoki K., Shikimi T., Hiranuma Y., Tomisugi Y., Ishikawa Y. (2002). Copper insertion facilitates water-soluble porphyrin binding to rA·rU and rA·dT base pairs in duplex RNA and RNA·DNA hybrids. Biochemistry.

[13] Ishikawa Y., Yamashita A., Uno T. (2001). Efficient photocleavage of DNA by cationic porphyrin-acridine hybrids with the effective length of diamino alkyl linkage. Chem. Pharm. Bull..

[14] Yamakawa N., Ishikawa Y., Uno T. (2001). Solution properties and photonuclease activity of cationic bis-porphyrins linked with a series of aliphatic diamines. Chem. Pharm. Bull..

[15] Ishikawa Y., Yamakawa N., Uno T. (2002). Potent DNA photocleavage by zinc(II) complexes of cationic bis-porphyrins linked with aliphatic diamine. Bioorg. Med. Chem..

[16] Aoki K., Ishikawa Y., Oyama M., Tomisugi Y., Uno T. (2003). Self-aggregation inhibits the photonuclease activity of porphyrins. Chem. Pharm. Bull..

[17] Villanueva A., Caggiari L., Jori G., Milanesi C. (1994). Morphological aspects of an experimental tumour photosensitized with a meso-substituted cationic porphyrin. J. Photochem. Photobiol. B..

[18] Mikami-Terao Y., Akiyama M., Yuza Y., Yanagisawa T., Yamada O., Yamada H. (2008). Antitumor activity of G-quadruplex-interactive agent TMPyP4 in K562 leukemic cells. Cancer Lett..

[19] Ishikawa Y., Yamakawa N., Uno T. (2007). Synthetic control of interchromophoric interaction in cationic bis-porphyrins toward efficient DNA photocleavage and singlet oxygen production in aqueous solution. Bioorg. Med. Chem..

[20] Coll M., Frederick C.A., Wang A.H., Rich A. (1987). A bifurcated hydrogen-bonded conformation in the d(A·T) base pairs of the DNA dodecamer d(CGCAAATTTGCG) and its complex with distamycin. Proc. Natl. Acad. Sci. U.S.A..

[21] (2005). MOE (Molecular Operating Environment).

[22] Tjahjono D.H., Akutsu T., Yoshioka N., Inoue H. (1999). Cationic porphyrins bearing diazolium rings: synthesis and their interaction with calf thymus DNA. Biochim. Biophys. Acta.

[23] Kuroda R., Tanaka H. (1994). DNA-porphyrin interactions probed by induced CD spectroscopy. J. Chem. Soc. Chem Commun..

[24] Pabo C.O., Sauer R.T. (1984). Protein-DNA recognition. Annu. Rev. Biochem..

